# Emerging Roles of MHC Class I Region-Encoded E3 Ubiquitin Ligases in Innate Immunity

**DOI:** 10.3389/fimmu.2021.687102

**Published:** 2021-06-10

**Authors:** Xiuzhi Jia, Chunyuan Zhao, Wei Zhao

**Affiliations:** Department of Pathogenic Biology, School of Basic Medical Science, Cheeloo College of Medicine, Shandong University, Jinan, China

**Keywords:** MHC class I region, E3 ubiquitin ligases, innate immunity, post-translational modifications, autoimmune diseases

## Abstract

The major histocompatibility complex (MHC) class I (MHC-I) region contains a multitude of genes relevant to immune response. Multiple E3 ubiquitin ligase genes, including tripartite motif 10 (*TRIM10*), *TRIM15*, *TRIM26*, *TRIM27*, *TRIM31*, *TRIM38*, *TRIM39*, *TRIM40*, and RING finger protein 39 (*RNF39*), are organized in a tight cluster, and an additional two TRIM genes (namely *TRIM38* and *TRIM27*) telomeric of the cluster within the MHC-I region. The E3 ubiquitin ligases encoded by these genes possess important roles in controlling the intensity of innate immune responses. In this review, we discuss the E3 ubiquitin ligases encoded within the MHC-I region, highlight their regulatory roles in innate immunity, and outline their potential functions in infection, inflammatory and autoimmune diseases.

## Introduction

Innate immunity is the first line of defense against invading pathogens and cancers. A variety of germline-encoded pattern recognition receptors (PRRs) recognize conserved structures present in pathogenic microorganisms (termed as pathogen-associated molecular patterns) and danger signals (termed as damage-associated molecular patterns), in turn initiating innate immune responses. The different types of PRRs include Toll-like receptors (TLRs), retinoic acid-inducible gene-I (RIG-I)-like receptors (RLRs), and cytosolic DNA sensors [e.g., cyclic GMP-AMP synthase (cGAS)]. These PRRs transduce activation signals by recruiting cellular adaptors including myeloid differentiation factor 88, Toll/IL-1 receptor (TIR) domain-containing adapter inducing interferon (IFN)-β (TRIF), mitochondrial antiviral signaling protein (MAVS), and stimulator of interferon genes (STING). These activation signals then activate the transcription factors nuclear factor kappa-B (NF-κB) and interferon regulatory factor 3 (IRF3), leading to the expression of proinflammatory cytokines and type I IFNs (1–3).

**Figure 1:**
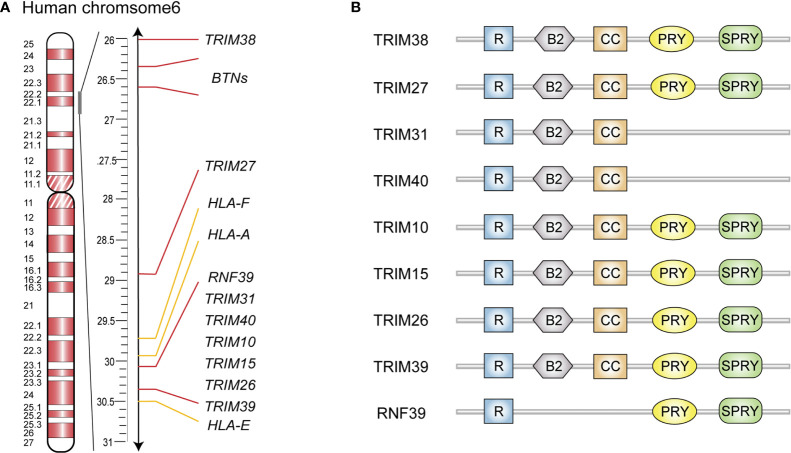
Schematic representation of gene clusters and structure domain of MHC-I region encoded E3 ubiquitin ligases. **(A)** Gene cluster of human MHC-I region. **(B)** Schematic diagram of structure domain of MHC-I region encoded E3 ubiquitin ligases. R, RING finger; B, B-box; CC, coiled-coil.

Optimal activation of innate immunity is crucial for the elimination of invading pathogens and mutant cells, as well as for the maintenance of immune homeostasis. A magnitude of sophisticated strategies have been developed by our body to manipulate the intensity of innate immune response, including epigenetic regulation and post-translational modifications (PTMs) of key immune signaling adaptors (4). Ubiquitination is an important PTM that is dynamically controlled by multiple E3 ubiquitin ligases and deubiquitinases, and it has been implicated in innate immune response (5). Recently, a series of E3 ubiquitin ligases encoded within the MHC-I region (**Figure 1**) have been reported as important regulators of innate immunity. In this review, we will introduce the MHC-I region genes encoded E3 ubiquitin ligases, highlight their regulatory roles in innate immunity and potential functions in infection, inflammatory and autoimmune diseases.

## Cluster of E3 Ubiquitin Ligases Genes in the MHC-I Region

MHC-I region contains a large number of immune-related genes, which are often polymorphic and closely linked as a result of their genomic proximity (6). In addition, many of these genes are associated with infections and autoimmune diseases, such as rheumatoid arthritis (RA) and systemic lupus erythematosus (SLE) (6). The human MHC-I genomic region locates on chromosome 6p21.33-6p22.2, known as human leukocyte antigen (HLA) class I region which contains HLA gene loci and several non-HLA gene clusters (7). Several E3 ubiquitin ligase genes in this region are organized in a tight cluster from HLA-E to HLA-A (**Figure 1A**), and they comprise six TRIM family members (including *TRIM10, TRIM15, TRIM26, TRIM31, TRIM39* and *TRIM40*) as well as *RNF39* (8). Two additional TRIM genes (namely *TRIM27* and *TRIM38*) are found telomeric from this cluster and near to the butyrophilin genes (8). In the mouse genome, *Trim10, Trim15, Trim26, Trim31, Trim39, Trim40* and *Rnf39* are located on chromosome 17 in the B1 region within the histocompatibility(H)2-I genetic group, while *Trim27* and *Trim38*, are located on chromosome 13 in the A3.1 region.

## Structure of MHC-I Region-Encoded E3 Ubiquitin Ligases

Most MHC-I region encoded E3 ubiquitin ligases are members of the TRIM protein family and possess RING finger, B-box 2, and coiled-coil (CC) domain (**Figure 1B**) (9, 10). The N-terminal RING finger domain confers the E3 ubiquitin ligase activity, which is essential for TRIMs to exert their antiviral effects and regulate innate immune signaling pathways (11). Specifically, the RING domain functions by recognizing E2 ubiquitin-conjugating enzymes *via* zinc finger motifs, subsequently transferring the ubiquitins or ubiquitin-like proteins to their substrates (11). The second signature sequence of MHC-I region encoded TRIM proteins is the B-box 2 domain, which also exhibits zinc-finger motifs similar to RING domain. Currently, the unified function of B-box 2 remains unclear, but there is evidence that it can potentiate the ability of TRIM5α to mediate human immunodeficiency virus 1 (HIV-1) restriction; lead to higher-order self-assembly of TRIM5α; and offer an E2 binding site resembling RING, which endows E3 ligase activity in some TRIMs lacking a RING domain (12–14). Following the B-box 2 domain is the CC domain, a typical hyper-secondary structure that can assemble with other CC structures to mediate homo- or hetero- oligomeric interactions among TRIM proteins (15). This oligomerization promotes the generation of high-molecular-mass complexes that are compartmentalized either in distinct cellular compartments such as nuclear bodies (PML/TRIM19) or microtubules (MID1/TRIM18) (16). In addition, structural analyses of several TRIM CC dimers have indicated that they are formed by antiparallel dimeric architecture, which places the RING and B-box domains on opposite sides of the CC domain. This architecture permits the dimerization of RING domains in some cases, endowing E3 ligase activity to some TRIM proteins (15). Furthermore, TRIM10, TRIM15, TRIM26, TRIM27, TRIM38, TRIM39 and RNF39 share a C-terminal PRY-SPRY domain (**Figure 1B**) that binds with high specificity to a diverse set of substrates, including peptides and proteins, and even RNA molecules (17, 18).

## Emerging Roles of MHC-I Region Encoded E3 Ubiquitin Ligases in Innate Immunity

**Figure 2:**
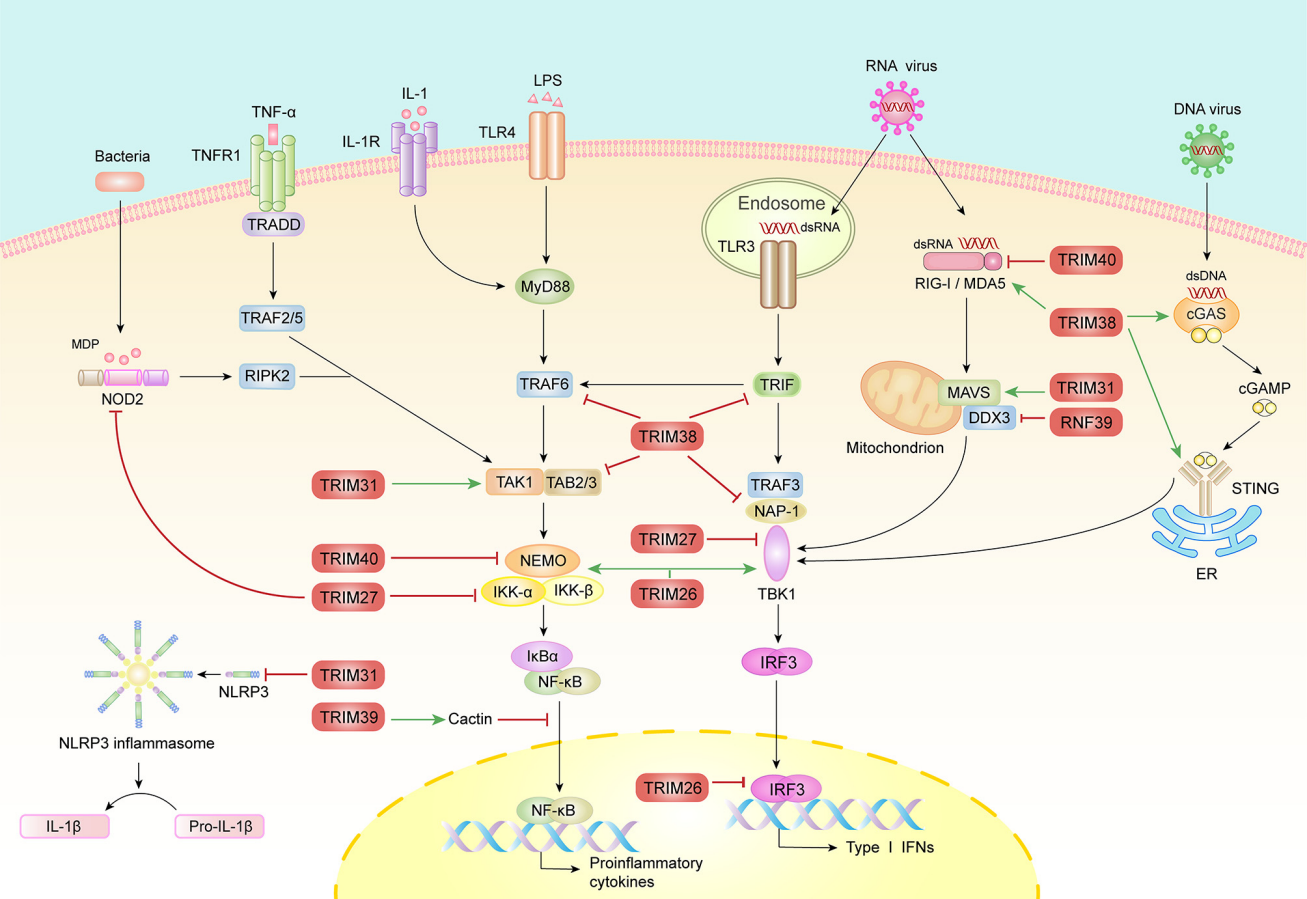
The roles of MHC-I region encoded E3 ubiquitin ligases in innate immunity. Multiple PRRs including TLRs, RLRs, NLRs and DNA sensors detect the invasion of pathogen and trigger downstream complex signaling pathways that culminate in the activation of transcription factor, IRF3 and NF-κB, resulting in the induction of type I IFNs and proinflammatory factors. Conversely, inflammatory cytokines, such as TNFα and IL-1β further active NF-κB signaling pathway. During these processes, E3 ubiquitin ligases catalyze diverse molecules including polyubiquitin, SUMO and Nedd8 to adaptors to ensure optimal activation or timely turned-off of signals. This figure has displayed the positive (green arrows) and negative (red lines) function of the MHC-I region encoded E3 ubiquitin ligases in PRR-mediated innate immune signaling pathways.

Increasing evidences indicate that the MHC-I region encoded E3 ubiquitin ligase genes possess moderate levels of polymorphism, play regulatory roles in innate immune responses (**Figure 2**), and their expressions are associated with a variety of autoimmune diseases (8).

### TRIM38

TRIM38 may be involved in the development of various autoimmune diseases and generally in the innate immune response. In one study, the presence of autoantibodies to TRIM38 significantly correlated with disease severity in patients with primary Sjögren’s syndrome (SS, a disease in which circulating autoantibodies react with multiple cellular proteins to cause glandular dysfunction) (19). Similarly, in patients with dermatomyositis (DM, an autoimmune connective tissue disease characterized by erythema in the eyes and hands, and weakness in the proximal muscles), skin and muscle biopsy analyses showed that *TRIM38* gene expression was upregulated (20). In fact, TRIM38 is an intriguing regulator of innate immunity (21). As a negative regulator, TRIM38 mediates lysine 48 (K48)-linked polyubiquitination of TNF receptor associated factor 6, TRIF and NF-κB-activating kinase-associated protein 1 to promote their proteasomal degradation, resulting in the inhibition of TLR and RLR pathways (22–24). In addition, TRIM38 facilitates the lysosome-dependent degradation of TAK1-binding protein 2 (TAB2) in TNF- and IL-1β-triggered signaling, independent of its E3 ubiquitin ligase activity; however, the specific mechanism remains to be explored (25). Notably, the interaction between TRIM38 and TAB2/3 is weakened in RA, resulting in an excess expression of TAB2/3 and proinflammatory cytokines, indicating the essential roles of TRIM38 in modulating autoimmune disease severity (26).

Besides its E3 ubiquitin ligase activity, TRIM38 has also been identified as an E3 small ubiquitin-like modifier (SUMO) ligase and mediates the SUMOylation of RIG-I and MDA5 (27). First, TRIM38 catalyzes the SUMOylation of RIG-I and MDA5, subsequently inhibiting K48-linked ubiquitination and degradation through steric hindrance both in rest and infection states (27). Second, this SUMOylation of RIG-I and MDA5 at K96/K889 and K43/K865 mediated by TRIM38 facilitates PP1-mediated dephosphorylation and K63-linked polyubiquitination of RIG-I/MDA5, leading to RLRs activation (27). Third, the SUMOylation of RIG-I/MDA5 impaired and the K48-linked ubiquitination increased in the late phase of viral infection, restricting both the intensity and duration of RLRs activation (27). This may also explain why in a different study, Enterovirus 71, another RNA virus, escaped immune surveillance through promoting degradation of TRIM38 (28). In DNA-sensing signaling pathways, TRIM38 also regulates the activation and expression of both cGAS and STING by mediating their SUMOylation by different mechanisms (29). Thus, TRIM38 may be the key in establishing an efficient antiviral state in the early phase of viral infection, while also terminating the activation of RLRs and cGAS/STING in the late phase. These results indicate that TRIM38 is a multifunctional molecule in innate immunity, and even affects the same pathway through different mechanisms (such as RLRs).

### TRIM27

TRIM27, also known as RET finger protein, plays important regulatory roles in innate immune responses; its dysregulation may cause several inflammatory diseases. TRIM27 binds to NOD2 *via* its PRY-SPRY domain, subsequently promoting K48-linked polyubiquitination and degradation of NOD2 and leading to the inhibition of NF-κB signal (30). An immunohistological inspection indicated that when compared to healthy tissue, *TRIM27* expression appeared higher in tissue derived from patients with Crohn disease (CD), implying that *TRIM27* has a role in CD, which is a known NOD2-related inflammatory disease (30). Additionally, in a model of hepatic ischemia-reperfusion injury (IRI, which initiates from oxidative stress and inflammation caused by insufficient blood supply and subsequent reperfusion owing to trauma, resection or transplantation of the liver), TRIM27 alleviated liver damage and inflammation by suppressing the recruitment of TAK1 *via* TAB2/3 degradation (31). Moreover, TRIM27 interacts with multiple IKKs, including IKKα, IKKβ, IKKϵ and TBK1, to attenuate both NF-κB and IRF triggered IFN-β expression (32). Furthermore, TRIM27 promotes IL-6-induced STAT3 activation by mediating ubiquitination of protein inhibitor of activated STAT3, thereby aggravating psoriasis (a chronic inflammatory disease that predominantly affects the skin and joints), colitis, and colitis-associated cancer (33, 34). In addition, it has been reported that TRIM27 is a host restriction factor that suppresses the survival of *Mycobacterium tuberculosis* in macrophages by upregulating the NF-κB and JNK/p38 pathways (35). Another study showed that TRIM27 could serve as a potential biomarker for discriminating active tuberculosis from latent tuberculosis infection and healthy people (36).

Recently, TRIM27 was reported to promote K48-linked ubiquitination at Lys251/372 and protein degradation of TBK1 during Vesicular stomatitis virus (VSV) infection (37). Furthermore, TRIM27 facilitates hepatitis C virus (HCV) replication through inhibiting IRF3 and NF-κB pathway (38). However, TRIM27 possesses contrary roles in Herpes simplex virus type 1 (HSV-1), HIV-1 and N-tropic murine leukemia virus (N-MLV)-infected cells; the contrary effects of TRIM27 may be related to varying effects of TRIM27 upon the lifecycles of different viruses (39, 40). While TRIM27 may regulate different innate immune pathways to control a type I IFN response during RNA virus and DNA virus infection, its effect during retrovirus infection may depend on direct interaction with viral components. Moreover, considering its vital role in regulating host antiviral immune responses, TRIM27 turn out to be the evasion target of HSV-1. The ICP0 protein of HSV-1, which has its own RING domain and E3 ubiquitin ligase activity, has been shown to interact with TRIM27 to promote its polyubiquitination and degradation (41). These contradictory results suggest complex roles of TRIM27 in innate immunity.

### TRIM31

Single nucleotide polymorphisms (SNPs) of *TRIM31*, also called hemochromatosis candidate gene I, are associated with a variety of inflammatory diseases including inflammatory bowel disease (IBD, a chronic nonspecific inflammatory disease characterized by recurrent inflammation of the intestinal mucosa, comprising two main distinctive entities, ulcerative colitis and CD) and irritant contact dermatitis (ICD, a kind of skin inflammatory reaction after contact with exogenous irritants) (42, 43). Additionally, TRIM31 expression correlates with reduced risk of nasopharyngeal carcinoma associated with Epstein–Barr virus infection (44). These evidences suggest the potential roles of TRIM31 in antiviral immune responses and autoimmune disorders. A confirmatory study identified that TRIM31 suppressed NLR family pyrin domain containing 3 (NLRP3) inflammasome activation by promoting K48-linked ubiquitination and proteasomal degradation of NLRP3 (45). The study also showed that *Trim31* deficiency aggravates alum-induced peritonitis and attenuates the severity of DSS-induced colitis (45). Furthermore, TRIM31 reduces the risk of other NLRP3 inflammasome-associated diseases such as apical periodontitis (AP, an acute suppurative inflammation caused by endodontic microbial infections) and age-related macular degeneration (AMD, a major cause of blindness in the elderly in developed countries, induced by dysfunction of retinal pigment epithelial cells, which constitute the immune defense barrier of the macula) (46, 47). In addition, TRIM31 is involved in the development of sepsis and colorectal cancer through regulating the NF-κB signaling pathway (48, 49).

In addition to its role in inflammation, TRIM31 has been shown to modify multiple MAVS sites with K63-linked polyubiquitin, leading to its aggregation and thus the enhancement of IFN-I expression upon RNA virus infection (50). The PB1-F2 protein of avian influenza A (H7N9) virus, scaffold protein FAF1 and Rho family small guanosine triphosphatase Rac1 limit the interaction between MAVS and TRIM31, resulting in the inhibition of MAVS ubiquitination, aggregation, and activation (51–53). TRIM31 also triggers ubiquitination and degradation of the hepatitis B virus (HBV) component HBx and therefore plays a potential role in IFN-resistant HBV infection (54). Furthermore, while TRIM31 can inhibit HIV-1 entry, downregulation of endogenously expressed *TRIM 31* inhibits both HIV-1 and MLV release, suggesting that TRIM31 plays different roles at early and late stages of the retroviral lifecycle (40). Overall, these findings provide the possibility of TRIM31 as a potential antiviral drug target.

### TRIM40

Genome-wide association studies show strong association between genetic variants of *TRIM40* and common diseases. For example, one of *TRIM40* SNPs, *rs757262*, can balance the risk of developing different autoimmune diseases (55). Accordingly, several functional studies indicate that TRIM40 is a regulator of innate immunity. TRIM40 physically combines with Nedd8 to promote the neddylation of IKKγ, thereby preventing gastrointestinal neoplasia caused by chronic inflammation; reduces inflammation and liver injury in septic mice *via* attenuating the activation of TLR4 pathway; and suppresses RLRs pathway by promoting both K27- and K48-linked polyubiquitination of RIG-I and MDA5, thus enhancing their proteasomal degradation (56–58). As RIG-I and MDA5 are associated with autoimmune diseases including Aicardi-Goutières syndrome (AGS, a severe autoimmune encephalopathy caused by aberrant activation of the IFN-I axis), Singleton–Merten syndrome(SMS, a type I interferonopathy characterized by aortic calcifications, psoriasis, glaucoma and skeletal abnormalities) and type 1 diabetes (T1D), TRIM40 may attenuate the pathogenesis of autoimmune diseases by regulating the activation of RIG-I and MDA5 (59–61).

### TRIM26

TRIM26, also called RNF95 or ZNF173, exhibits polymorphisms associated with several autoimmune disorders including T1D and multiple sclerosis (MS, an autoimmune disease characterized by inflammatory demyelination of the central nervous system) and with nasopharyngeal carcinoma caused by viral disease (62–64). In antiviral immunity, TRIM26 mediates the K48-linked polyubiquitination and protein degradation of nuclear IRF3, attenuating the antiviral response (65). However, another study reported that TRIM26 actually enhanced innate immunity against RNA viruses, by recruiting NEMO to facilitate the interaction between TBK1 and MAVS (66). Furthermore, a genome-wide CRISPR-Cas9 screening identified TRIM26 as a critical HCV host factor, where it mediates K27-linked ubiquitination of HCV-encoded NS5B protein, enhances the interaction between NS5B-NS5A, and ultimately promotes HCV genome replication (67). Thus, as a key E3 ubiquitin ligase, TRIM26 plays multiple roles through catalyzing the conjugation of multiple ubiquitin chains to variety of substrates.

### RNF39

Emerging evidences indicate that RNF39 is a potential immune regulator. Genetic variants of *RNF39* are associated with a variety of viral diseases and autoimmune diseases, such as the progression of HIV-1 and Behcet’s disease (BD, a chronic systemic vasculitis resulting in ulcerative in the oral cavity and on the genitals, as well as inflammatory damage of the eyes) (68). The DNA methylation state of *RNF39* impacts autoimmune disorders, including MS, SLE and allergic rhinitis (AR, a delayed hypersensitivity nasal mucosa reaction to environmental allergens, caused by IgE mediated release of autacoids) and confers poor responsiveness to HBV vaccination (69–72). Furthermore, *RNF39* genetic variants are related to HIV-1 plasma viral loads, CD4^+^ T cell count, and the clinical course of HIV-1 infection (73–75). Besides its role in DNA viruses and retrovirus infection, RNF39 has also been identified as a feedback suppressor of RNA virus-induced signaling and antiviral immunity. RNF39 mediates K48-linked polyubiquitination and proteasomal degradation of DDX3X, a scaffold vital for the formation of the MAVS-TRAF3 complex, to subsequently inhibit the RLR pathway (76). *Rnf39* deficiency enhances RLR activation and inhibits RNA viral replication (76). Further studies will identify more immune targets of RNF39, which will help to explain the regulatory roles of RNF39 in antiviral and autoimmune responses.

### Other MHC-I Region Encoded E3 Ubiquitin Ligases

Several other MHC-I region-encoded E3 ubiquitin ligases have also been reported to regulate viral infection and innate immunity. SNPs in *TRIM15* show a correlation with lupus nephritis (LN, an autoimmune disease characterized by the hypersecretion of autoantibodies and deposition of immune complexes in the kidneys); this correlation varies significantly according to ethnicity (77). Furthermore, human TRIM15 interferes with the release of HIV-1 and MLV from cells (40). TRIM15 targets adaptors upstream of MAVS to potentiate RIG-I-mediated IFN-β production and suppress viral infection (78). TRIM39 stabilizes Cactin to attenuate TLR- and TNF-α-mediated RelA/p65 translocation, inhibiting the expression of IL-6 and IL-8 (79). SNP analyses indicate that the genetic variants of *TRIM39* are also associated with inflammatory diseases including psoriasis, and autoimmune diseases including BD and cutaneous lupus erythematosus (CLE, an autoimmune connective tissue disease with cutaneous lesion) (68, 80, 81). Another study found a differentially methylated site in the promoter region of TRIM39-RPP2 that was associated with IBD patients (82). Lastly, TRIM39 also regulates the type I IFN response to exert its antiviral functions (83). TRIM10 contributes to the restriction of HIV-1 entry and shows high correlation with the risk of developing MS, RA, LN, ankylosing spondylitis (AS, a chronic autoimmune-mediated arthritis that predominantly affects the axial skeleton and peripheral joints), autoimmune thyroid disease (AITD, various conditions caused by autoantibodies attacking the thyroid, including Hashimoto’s thyroiditis and Graves’ disease) and T1D, although its exact functions in these conditions remain unknown (40, 77, 84). In summary, the detailed mechanisms of action of these lesser-studied MHC-I region-encoded E3 ubiquitin ligases (including TRIM10, TRIM15 and TRIM39) need to be further investigated.

## Conclusion and Perspective

Many MHC-I region-encoded E3 ubiquitin ligases are highly polymorphic, participate in the regulation of inflammation and antiviral innate immunity, and play a key role in the interaction between virus and host. At present, the known mechanisms of action include ubiquitination and SUMOylation; however, more mechanisms will be elucidated with further study. Concordantly, these MHC-I region genes are related to a variety of inflammatory, viral and autoimmune diseases. Nonetheless, their exact roles in the development of these diseases remain largely unclarified. In addition, same E3 ubiquitin ligases may play contrary roles in different inflammatory diseases. So, how does the organism regulate the action of the same MHC-I region-encoded E3 ubiquitin ligase (such as TRIM26 and TRIM38) to exert multiple or even opposing activities in different pathways? Further studies with varied cell types and disease models using gene-deficient mice are required to answer these questions. Given the important role of MHC-I region-encoded E3 ubiquitin ligases in antiviral innate immunity, it is tempting to speculate that viruses have evolved immune escape mechanisms by manipulating TRIM proteins. In addition, as some viruses are known to participate in oncogenesis or cancer progression, whether these E3 ubiquitin ligases could regulate the development of virus-related cancer deserves further investigation.

In this review, we have focused on the roles of MHC-I region-encoded E3 ubiquitin ligases in controlling the intensity of innate immune responses; however, whether these MHC-I region-encoded E3 ubiquitin ligases could modulate adaptive immunity requires further investigation. Elucidating their specific functions and molecular details in immune regulation will help us to further understand the vital roles of MHC-I region genes in immunity and provide promising diagnostic and therapeutic targets for diseases characterized by aberrant activation of innate immunity.

## Author Contributions

XJ drafted the manuscript and figures, CZ and WZ edited the manuscript, WZ supervised and edited the figures. All authors contributed to the article and approved the submitted version.

## Funding

This work was supported by grants from the National Natural Science Foundation of China (81622030, 31870866 and 81901609). WZ is a Newton Advanced Fellow awarded by the Academy of Medical Sciences.

## Conflict of Interest

The authors declare that the research was conducted in the absence of any commercial or financial relationships that could be construed as a potential conflict of interest.
